# Supplementary material for: Attitudes toward COVID-19 and Other Vaccines: Comparing Parents to Other Adults, September 2022
Source: Vaccines (Basel). 2023 Nov 21;11(12):1735. doi: 10.3390/vaccines11121735 (PMC10748314; doi:10.3390/vaccines11121735)
Supplement: Supplementary file 1 [file vaccines-11-01735-s001.zip › vaccines-2674602-supplementary.pdf]

Table S1. STROBE Statement

	Item No.	Recommendation	Page No.	Relevant text from manuscript
**Title and abstract**	1	(*a*) Indicate the study's design with a commonly used term in the title or the abstract	1	In this cross-sectional study, we surveyed US adults in September 2022, immediately following authorization of updated bivalent COVID-19 boosters for adults, but before their authorization for children.
		(*b*) Provide in the abstract an informative and balanced summary of what was done and what was found	1	Vaccine attitudes of parents were compared to other adults. Fewer parents were up-to-date on COVID-19 vaccines than other adults (54% vs. 67%), even after adjusting for age, education, and race/ethnicity (Adjusted Odds Ratio: 0.58; 95% Confidence Interval: 0.45-0.76). More parents had concerns about COVID-19 vaccines' safety in children (67% vs. 58%; aOR: 1.59; 95%CI: 1.23-2.06) and vaccine ingredients (52% vs. 45%; aOR: 1.41; 95%CI: 1.09-1.81), and more parents perceived low COVID-19 disease severity (51% vs. 38%; aOR: 1.56; 95%CI: 1.22-2.01). Fewer parents supported COVID-19 vaccine school requirements (52% vs. 57%; aOR: 0.75; 95%CI: 0.58-0.97) and perceived high vaccine coverage among their friends (51% vs. 61%; aOR: 0.60; 95%CI: 0.46-0.78). However, three-quarters of parents intended their child receive all routinely recommended vaccines, whereas only half of adults intended to receive all routinely recommended vaccines themselves.
**Introduction**				
Background/rationale	2	Explain the scientific background and rationale for the investigation being reported	2	Although many parental vaccine concerns existed before the pandemic, and vaccine attitudes among US parents may have initially improved at the outset of the pandemic, overall the pandemic seems to have had a negative impact on vaccine confidence. Vaccines unfortunately became a polarizing political issue during the pandemic, which, along with pandemic fatigue, has increased hesitancy to receive COVID vaccines, and perhaps routine vaccines as well. Frequent collection and review of representative survey data is needed to understand trends in vaccine hesitancy, especially if the drops in routine vaccine coverage from the early pandemic are to be regained.
Objectives	3	State specific objectives, including any prespecified hypotheses	2	The main focus of this analysis was to compare the vaccine attitudes of parents to other adults, both for COVID-19 and for other recommended vaccines.
**Methods**				

Study design	4	Present key elements of study design early in the paper	2	A national panel survey was administered between September 1-12, 2022, in English and Spanish, using Ipsos KnowledgePanel, the largest probability-based online panel in the US. Ipsos uses address-based sampling techniques to recruit members to ensure the geodemographic composition of the panel mimics the adult US population. Stratified random selection, enrollment quotas, and survey weights ensured the sociodemographic distribution of our sample remained representative of the adult US population even while oversampling Hispanic and Black respondents by 50% to increase power to detect differences by race/ethnicity. We have successfully used Ipsos KnowledgePanel for related surveys previously. More detail on the methodology of this survey is described elsewhere. The Johns Hopkins Bloomberg School of Public Health Institutional Review Board considered this work to be public health surveillance and not human subject research.
Setting	5	Describe the setting, locations, and relevant dates, including periods of recruitment, exposure, follow-up, and data collection	2	A national panel survey was administered between September 1-12, 2022, in English and Spanish, using Ipsos KnowledgePanel, the largest probability-based online panel in the US.
Participants	6	(*a*) *Cohort study*—Give the eligibility criteria, and the sources and methods of selection of participants. Describe methods of follow-up<br>*Case-control study*—Give the eligibility criteria, and the sources and methods of case ascertainment and control selection. Give the rationale for the choice of cases and controls<br>*Cross-sectional study*—Give the eligibility criteria, and the sources and methods of selection of participants	2	Ipsos uses address-based sampling techniques to recruit members to ensure the geodemographic composition of the panel mimics the adult US population. Stratified random selection, enrollment quotas, and survey weights ensured the sociodemographic distribution of our sample remained representative of the adult US population even while oversampling Hispanic and Black respondents by 50% to increase power to detect differences by race/ethnicity.
		(*b*) *Cohort study*—For matched studies, give matching criteria and number of exposed and unexposed<br>*Case-control study*—For matched studies, give matching criteria and the number of controls per case		n/a

Variables	7	Clearly define all outcomes, exposures, predictors, potential confounders, and effect modifiers. Give diagnostic criteria, if applicable	2-3	One primary outcome of this survey was COVID-19 vaccination status. The survey began by asking respondents to identify themselves as either: (1) up-to-date (i.e., fully vaccinated and boosted); (2) vaccinated, but not up-to-date (e.g., have not gotten a booster yet); (3) not having received any COVID-19 vaccines; or (4) prefer not to say (a response only given by 3% of the sample and thus treated as missing).<br>This survey also measured attitudes about vaccines for both children and adults, including constructs such as perceived susceptibility to and severity of vaccine-preventable diseases and the importance of COVID-19 vaccines. Among those not yet up-to-date on COVID-19 vaccines nor intending to get up-to-date as soon as possible, respondents identified their concerns and other reasons for not vaccinating. Confidence in sources of COVID-19 information, cumulative COVID-19 disease prevalence (ever having COVID-19 disease), and self-reported influenza vaccination were captured. Trust in the Centers for Disease Control and Prevention (CDC) was measured using a 14-item scale, described elsewhere.[18,23]<br>Respondents were asked how many children (less than 18 years old) they had, and the age of each child. Parents of at least one preteen 11-12 years old were given additional survey items pertaining to these children. Parents of children under 5 years old (who did not also have a child 11-12 years old) were given similar items pertaining to their child(ren) under 5 years old. These two age ranges were targeted to reflect the ages at which most vaccines are recommended according to the CDC schedule: most vaccines are given in the first five years of life, including vaccines required for kindergarten by law in many states (e.g., DTaP, polio, MMR, varicella, hepatitis B), though three vaccines (HPV, Tdap, meningococcal) are not recommended until 11 years of age.[24]<br>Survey items measured parental intentions to get their children the vaccines recommended for their age group, confidence in the safety of these vaccines, self-efficacy (confidence they could get their child vaccinated), perceived vaccine knowledge, and specific vaccine concerns. Adults who were not parents of children 0-5 or 11-12 years old were given similar survey items but focused on adult vaccines recommended for themselves (since these adults were likely due the same number or more vaccines than their children), and were split by those 18-50 years old versus over 50 years old, again to reflect the vaccine schedule (e.g., the herpes zoster vaccine is only recommended for adults over 50).[25] In essence, these four age groups of interest

				(parents of children 0-5, parents of preteens 11-12, adults 18-50, adults 50+) were made mutually exclusive with each respondent only receiving the additional survey items for one of the four age groups, with priority given to the smallest group, to avoid redundancy and reduce survey length while maintaining power for precise estimates within each group.<br>Gender, age, race/ethnicity, education, income, employment status, metropolitan statistical area (MSA), region, and political affiliation were among the sociodemographic characteristics available for all respondents. Choices in survey content were influenced by the Health Belief Model and the Social Ecological Model.
Data sources/ measurement	8*	For each variable of interest, give sources of data and details of methods of assessment (measurement). Describe comparability of assessment methods if there is more than one group	3	Design weights were adjusted using a raking procedure to imitate the US adult population. Hispanic and Black respondents were oversampled to increase power for stratified analyses, but down-weighted to reflect their proportion in the population (Table 1). Further details on this weighting technique have been published elsewhere. Sociodemographic characteristics (Table 2) and vaccine attitudes (Table 3) were cross-tabulated against parent status, and vaccine attitudes were also cross-tabulated against oldest child age (comparing older to younger) (Appendix A). Odds ratios were calculated. General vaccine attitudes and safety concerns were cross-tabulated against general vaccine intentions (Table 4). Likert and other scale response options were dichotomized to reflect affirmative versus negative (e.g., agree vs. disagree, important vs. not important) to facilitate straightforward analyses and interpretation.<br>Standard errors for weighted proportions were calculated using Taylor-linearized variance estimation. P-values for cross-tabulations were calculated using the Pearson chi-squared proportion test ($\alpha = 0.05$). Bivariate odds ratios were calculated using generalized logistic binomial regression with a logit link function. In Table 2, simple logistic regressions featured parent status as the dependent variable and other sociodemographic characteristics as independent variables. In Table 3, multiple logistic regressions featured affirmative survey responses as the dependent variable and parent status as the main in-dependent variable, with the sociodemographic characteristics significantly associated with parent status in Table 2 included as additional independent variables to adjust for potential confounding. Data were analyzed using Stata statistical software (version 16).

Bias	9	Describe any efforts to address potential sources of bias	3	In Table 3, multiple logistic regressions featured affirmative survey responses as the dependent variable and parent status as the main in-dependent variable, with the sociodemographic characteristics significantly associated with parent status in Table 2 included as additional independent variables to adjust for potential confounding.
Study size	10	Explain how the study size was arrived at	3	The sample size for this survey was chosen to approximate the sample size of our previous related KnowledgePanel surveys, which were well powered to demonstrate attitudinal associations with vaccine status and intentions

Continued on next page

Quantitative variables	11	Explain how quantitative variables were handled in the analyses. If applicable, describe which groupings were chosen and why	3	Sociodemographic characteristics (Table 2) and vaccine attitudes (Table 3) were cross-tabulated against parent status, and vaccine attitudes were also cross-tabulated against oldest child age (comparing older to younger) (Appendix A). Odds ratios were calculated. General vaccine attitudes and safety concerns were cross-tabulated against general vaccine intentions (Table 4). Likert and other scale response options were dichotomized to reflect affirmative versus negative (e.g., agree vs. disagree, important vs. not important) to facilitate straightforward analyses and interpretation.<br>Standard errors for weighted proportions were calculated using Taylor-linearized variance estimation. P-values for cross-tabulations were calculated using the Pearson chi-squared proportion test ($\alpha = 0.05$). Bivariate odds ratios were calculated using generalized logistic binomial regression with a logit link function. In Table 2, simple logistic regressions featured parent status as the dependent variable and other sociodemographic characteristics as independent variables. In Table 3, multiple logistic regressions featured affirmative survey responses as the dependent variable and parent status as the main in-dependent variable, with the sociodemographic characteristics significantly associated with parent status in Table 2 included as additional independent variables to adjust for potential confounding.
Statistical methods	12	(*a*) Describe all statistical methods, including those used to control for confounding<br>(*b*) Describe any methods used to examine subgroups and interactions<br>(*c*) Explain how missing data were addressed<br>(*d*) *Cohort study*—If applicable, explain how loss to follow-up was addressed<br>*Case-control study*—If applicable, explain how matching of cases and controls was addressed<br>*Cross-sectional study*—If applicable, describe analytical methods taking account of sampling strategy<br>(*e*) Describe any sensitivity analyses	3	Design weights were adjusted using a raking procedure to imitate the US adult population. Hispanic and Black respondents were oversampled to increase power for stratified analyses, but down-weighted to reflect their proportion in the population (Table 1). Further details on this weighting technique have been published elsewhere.<br>Sociodemographic characteristics (Table 2) and vaccine attitudes (Table 3) were cross-tabulated against parent status, and vaccine attitudes were also cross-tabulated against oldest child age (comparing older to younger) (Appendix A). Odds ratios were calculated. General vaccine attitudes and safety concerns were cross-tabulated against general vaccine intentions (Table 4). Likert and other scale response options were dichotomized to reflect affirmative versus negative (e.g., agree vs. disagree, important vs. not important) to facilitate straightforward analyses and interpretation.<br>Standard errors for weighted proportions were calculated using Taylor-linearized variance estimation. P-values for cross-tabulations were calculated using the Pearson chi-squared proportion test ($\alpha = 0.05$). Bivariate odds ratios were calculated using generalized logistic binomial regression with a logit link function. In Table 2, simple

				logistic regressions featured parent status as the dependent variable and other sociodemographic characteristics as independent variables. In Table 3, multiple logistic regressions featured affirmative survey responses as the dependent variable and parent status as the main in-dependent variable, with the sociodemographic characteristics significantly associated with parent status in Table 2 included as additional independent variables to adjust for potential confounding.
**Results**				
Participants	13*	(a) Report numbers of individuals at each stage of study—eg numbers potentially eligible, examined for eligibility, confirmed eligible, included in the study, completing follow-up, and analysed	4	The survey was fielded among 5323 panel members. Of these, 2787 (52%) completed the survey, of which 2,561 qualified for the study (based on eligibility criteria and survey quotas).
		(b) Give reasons for non-participation at each stage		n/a
		(c) Consider use of a flow diagram		n/a
Descriptive data	14*	(a) Give characteristics of study participants (eg demographic, clinical, social) and information on exposures and potential confounders	4	Unweighted and weighted sociodemographic characteristics and vaccination status of the study population are presented in Table 1. Weighted data are generalizable to the adult population of the US.
		(b) Indicate number of participants with missing data for each variable of interest		n/a
		(c) *Cohort study*—Summarise follow-up time (eg, average and total amount)		
Outcome data	15*	*Cohort study*—Report numbers of outcome events or summary measures over time		
		Case-control study—Report numbers in each exposure category, or summary measures of exposure		
		Cross-sectional study—Report numbers of outcome events or summary measures	5	Nearly 30% of the weighted sample had at least one child less than 18 years old (Table 2).
Main results	16	(*a*) Give unadjusted estimates and, if applicable, confounder-adjusted estimates and their precision (eg, 95% confidence interval). Make clear which confounders were adjusted for and why they were included	6, 8	Parents were less likely to be up-to-date on their COVID-19 vaccines than other adults (54% vs. 67%), even after adjusting for age, education, and race/ethnicity (Adjusted Odds Ratio: 0.58; 95% Confidence Interval: 0.45-0.76) (Table 3). Parents were also more likely to report ever having COVID-19 disease (54% vs. 40%; aOR: 1.50; 95%CI: 1.17-1.93).

		Parents were less likely to report that most of their friends had gotten vaccinated against COVID-19 than other adults (51% vs. 61%; aOR: 0.60; 95%CI: 0.46-0.78) or support a requirement for children to be vaccinated against COVID-19 to attend school (52% vs. 57%; aOR: 0.75; 95%CI: 0.58-0.97). Parents were more likely to report concerns about the safety of COVID-19 vaccines in children (67% vs. 58%; aOR: 1.59; 95%CI: 1.23-2.06) or vac-cine ingredients (52% vs. 45%; aOR: 1.41; 95%CI: 1.09-1.81). Parents were also more likely to believe it better for children to develop immunity to COVID-19 by getting sick rather than by getting a shot (45% vs. 33%; aOR: 1.56; 95%CI: 1.20-2.02) or that COVID-19 in children is no worse than a cold or the flu (51% vs. 38%; aOR: 1.56; 95%CI: 1.22-2.01).
	(*b*) Report category boundaries when continuous variables were categorized	See Table 2
	(*c*) If relevant, consider translating estimates of relative risk into absolute risk for a meaningful time period	n/a

Continued on next page

Other analyses	17	Report other analyses done—eg analyses of subgroups and interactions, and sensitivity analyses	8-9	Three-quarters (76%) of parents of preteens 11-12 years old intended for that child to receive all recommended vaccines in adolescence; 22% were unsure or intended to skip some adolescent vaccines, and 2% intended to skip all adolescent vaccines (Table 4). Four-fifths (80%) of parents of children under 5 years old (who did not also have a child 11-12 years old) intended for that child to receive all recommended vaccines in childhood; 18% were unsure or intended to skip some childhood vaccines, and 2% intended to skip all childhood vaccines. In contrast, two-fifths (41%) of adults 18-50 years old (who did not have a child 0-5 or 11-12 years old) intended to receive all vaccines recommended for young adults, while 41% were unsure or intended to skip some adult vaccines, and 18% intended to receive no adult vaccines; 54% of adults over 50 years old (who did not have a child 0-5 or 11-12 years old) intended to receive all vaccines recommended for older adults, while 38% were unsure or intended to skip some adult vaccines, and 9% intended to receive no adult vaccines.
**Discussion**				
Key results	18	Summarise key results with reference to study objectives	13	Parents of children were less likely to be up-to-date on their COVID-19 vaccines than other adults and more likely to report ever having COVID-19 disease. Parents were also more likely to report concerns about vaccine safety and ingredients and perceive low COVID-19 disease severity than other adults, and were less likely to support COVID-19 vaccine school requirements, and to perceive high vaccine coverage among their friends.
Limitations	19	Discuss limitations of the study, taking into account sources of potential bias or imprecision. Discuss both direction and magnitude of any potential bias	15	The CDC now considers everyone 6 years and older to be "up-to-date" if they have received an updated COVID-19 vaccine (children 6 months to 5 years may need multiple doses to be up to date, but at least one dose must be the updated COVID-19 vaccine).[8,42] However, when this survey was administered in September 2022, "up-to-date" was de-fined as fully vaccinated (with a primary series) and boosted. No specification was made regarding if the booster must be the most recent version, as the bivalent booster was not yet widely available, having just been authorized.[6] Thus we are unable to differentiate be-tween booster versions in our data and assume our measure of "up-to-date" refers largely to the now obsolete monovalent booster. However, our survey was also well timed to capture the proportion of US parents and other adults who had received a monovalent booster dose just before it was replaced by the updated bivalent version.

				Another limitation of our study is its reliance on data from one point in time rather than over an extended period. Our data are also subject to the limitations of self-reporting. However, most analyses of COVID-19 vaccine attitudes do not cover routine vaccines, nor have they focused on parents in particular, as ours does. Furthermore, many other analyses of COVID-19 vaccine attitudes have not been subjected to peer review, and their in-ternal and external validity varies widely. A strength of this analysis is its use of high-quality data from a well-established nationally representative panel.
Interpretation	20	Give a cautious overall interpretation of results considering objectives, limitations, multiplicity of analyses, results from similar studies, and other relevant evidence	15	Immunization programs must reemphasize and sustain efforts to support parents as they make vaccine decisions both for themselves and their children. The public health community should ensure pediatric providers have the resources needed to discuss the risks and benefits of vaccines with their patients' parents, especially as new vaccines are authorized, and recommendations are updated.
Generalisability	21	Discuss the generalisability (external validity) of the study results	15	A strength of this analysis is its use of high-quality data from a well-established nationally representative panel.
**Other information**				
Funding	22	Give the source of funding and the role of the funders for the present study and, if applicable, for the original study on which the present article is based	15	Funding: This work was supported in part by a research grant from the Investigator-Initiated Studies Program of Merck Sharp & Dohme LLC. The opinions expressed in this paper are those of the authors and do not necessarily represent those of Merck Sharp & Dohme LLC. Merck Sharp & Dohme LLC was not involved in any aspects of the study, including study design; data collection, analyses, and interpretation; writing of the research article; and in the decision to submit the article for publication.

*Give information separately for cases and controls in case-control studies and, if applicable, for exposed and unexposed groups in cohort and cross-sectional studies.

Note: An Explanation and Elaboration article discusses each checklist item and gives methodological background and published examples of transparent reporting. The STROBE checklist is best used in conjunction with this article (freely available on the Web sites of PLoS Medicine at http://www.plosmedicine.org/, Annals of Internal Medicine at http://www.annals.org/, and Epidemiology at http://www.epidem.com/). Information on the STROBE Initiative is available at www.strobe-statement.org.

Table S2. Vaccine Attitudes and Values of Parents of Older Children (at least 11 Years of Age) versus Parents of Younger Children (0-10 Years of Age)

Survey Items	All Parents (%)[a]	Age of Oldest Child (%)[b]		p-value[c]	aOR (95%CI)[d]
		0-10	11+		
**All Parents of Children <18 Years of Age**	100	43	57		
**Vaccination and Disease Status**					
Vaccinated against flu within the past year	52	57	49	0.10	**0.48 (0.32-0.72)**
Vaccinated against COVID (at least one dose)	82	79	84	0.24	0.88 (0.49-1.59)
Up-to-date on COVID vaccines	54	47	58	**0.02**	0.93 (0.60-1.42)
Ever knowingly had COVID disease	55	54	55	0.85	0.89 (0.59-1.32)
**Scales**					
Trust in the Centers for Disease Control and Prevention (CDC)	67	64	68	0.37	0.91 (0.59-1.41)
**Confidence in sources for information about COVID-19**					
My doctor	87	88	85	0.43	0.52 (0.27-1.00)
My local or state health department	74	77	71	0.20	**0.63 (0.39-0.99)**
Scientists and doctors from the CDC	72	75	69	0.23	0.71 (0.44-1.12)
The Surgeon General	70	72	68	0.33	0.74 (0.47-1.16)
Scientists and doctors from universities	75	77	74	0.49	0.77 (0.47-1.25)
Dr. Anthony Fauci from the National Institutes of Health	61	64	59	0.26	0.68 (0.44-1.04)
Dr. Rochelle Walensky, Director of the CDC	62	64	60	0.44	0.76 (0.49-1.16)
Dr. David Satcher, Morehouse School of Medicine, Former CDC Director and Surgeon General	62	62	62	0.98	0.88 (0.57-1.35)
My religious leader	32	32	33	0.85	1.08 (0.69-1.68)
Other non-medical people in my community that I trust	31	33	30	0.45	0.82 (0.50-1.34)
What I see on the news	32	28	34	0.19	1.17 (0.74-1.87)
What I see on social media (Facebook, Twitter, etc.)	15	14	16	0.60	1.43 (0.78-2.59)
**Agreement with COVID-19 Likert Scale Items (for Adults)**					
I worry I may accidentally spread COVID-19 to my family members in the next six months.	35	33	36	0.59	1.03 (0.69-1.53)
I worry I may accidentally spread COVID-19 to my friends, neighbors, or co-workers in the next six months.	33	33	33	0.95	0.96 (0.63-1.47)
If I get COVID-19, I think it will be severe.	15	15	15	0.95	1.42 (0.79-2.53)
COVID-19 vaccines are important to stopping the spread of infection in the US.	71	70	71	0.75	0.82 (0.53-1.28)
COVID-19 vaccines are important to helping the US get back to a normal life.	69	66	71	0.20	0.96 (0.61-1.51)

Most or all of my family members have gotten vaccinated against COVID-19.	63	61	64	0.63	0.76 (0.49-1.16)
Most or all of my friends have gotten vaccinated against COVID-19.	51	50	51	0.86	**0.59 (0.39-0.90)**
If my main doctor were to recommend that I take the COVID-19 vaccine, I'd be likely to take it.	47	42	51	0.22	1.12 (0.56-2.23)
If a close family member were to recommend that I take the COVID-19 vaccine, I'd be likely to take it.	37	33	40	0.29	1.23 (0.62-2.43)
If my close friends were to recommend that I take the COVID-19 vaccine, I'd be likely to take it.	33	30	34	0.53	1.19 (0.57-2.48)
I feel knowledgeable about the COVID-19 vaccine.	72	72	73	0.73	0.78 (0.50-1.23)
I'd like to get more information on COVID-19 vaccines.	30	35	27	**0.05**	0.82 (0.54-1.24)
**Agreement with COVID-19 Likert Scale Items (for Children)**					
COVID-19 can be a serious disease for some children.	83	86	80	0.13	**0.58 (0.34-0.96)**
I am concerned about the safety of COVID-19 vaccine in children.	67	71	65	0.16	0.87 (0.58-1.32)
Vaccinating children against COVID-19 is important to end the pandemic and get back to normal.	61	57	64	0.10	1.14 (0.75-1.74)
It is better for children to develop immunity to COVID-19 by getting sick rather than by getting a shot.	46	47	45	0.61	1.09 (0.72-1.63)
COVID-19 in children is no worse than a cold or the flu.	51	49	53	0.41	1.44 (0.97-2.16)
I would support a requirement for children to be vaccinated against COVID-19 to attend school.	52	50	54	0.38	0.97 (0.65-1.47)
My child(ren)'s doctor recommended that my child(ren) be vaccinated against COVID-19 once authorized by the FDA.	67	60	73	**0.01**	1.29 (0.83-2.02)
If not: I would feel more comfortable giving my child(ren) a COVID-19 vaccine if my child(ren)'s doctor recommended it.	27	35	18	**0.02**	**0.44 (0.21-0.95)**
I would feel more comfortable giving my child(ren) a COVID-19 vaccine that was fully approved for children by the FDA (instead of just authorized for emergency use).	66	68	64	0.45	0.79 (0.51-1.22)
**Agreement with General Vaccine Likert Scale Items**					
I am confident in the safety of vaccines.	79	80	78	0.74	0.71 (0.41-1.23)
I do not trust a vaccine unless it has already been safely given to millions of other people.	51	56	48	0.09	0.87 (0.58-1.30)
I am concerned about some of the ingredients in vaccines.	52	47	55	0.09	**1.54 (1.02-2.34)**
Vaccine recommendations from the Centers for Disease Control and Prevention (CDC) are a good fit for me.	75	79	72	0.07	0.61 (0.36-1.03)
I am concerned that the government and drug companies experiment on people like me.	47	49	46	0.49	1.02 (0.68-1.53)
The benefits of vaccines are much bigger than their risks.	79	80	78	0.66	0.75 (0.43-1.31)

Red text indicates survey items reflecting negative vaccine attitudes

[a] Column percentages (of total sample N=2561), weighted according to survey weights to achieve national representativeness

[b] Column percentages (of age of oldest child) (except for first row "All Parents of Children <18 Years of Age", which is a row percentage), weighted according to survey weights to achieve national representativeness

[c] Using the Pearson chi-square test at significance level of alpha=5%; bold indicates statistical significance ($p<0.05$)

[d] Adjusted Odds Ratio (95% Confidence Interval) of reporting agreement with the survey item in each row comparing parents of older children (oldest child at least 11 years of age) to parents of younger children (0-10 years of age), adjusted for parent age; bold indicates statistical significance ($p<0.05$)
